# Genome-Wide Knockout Screen Identifies Human Sialomucin CD164 as an Essential Entry Factor for Lymphocytic Choriomeningitis Virus

**DOI:** 10.1128/mbio.00205-22

**Published:** 2022-05-03

**Authors:** Jamin Liu, Kristeene A. Knopp, Elze Rackaityte, Chung Yu Wang, Matthew T. Laurie, Sara Sunshine, Andreas S. Puschnik, Joseph L. DeRisi

**Affiliations:** a Department of Biochemistry and Biophysics, University of California, San Francisco, San Francisco, California, USA; b University of California, Berkeley-University of California, San Francisco Graduate Program in Bioengineering, University of California, San Francisco, San Francisco, California, USA; c Chan Zuckerberg Biohub, San Francisco, California, USA; University of Wisconsin-Madison; St. Jude Children's Research Hospital

**Keywords:** CD164, CRISPR screen, lymphocytic choriomeningitis virus, viral entry

## Abstract

Lymphocytic choriomeningitis virus (LCMV) is a well-studied mammarenavirus that can be fatal in congenital infections. However, our understanding of LCMV and its interactions with human host factors remains incomplete. Here, host determinants affecting LCMV infection were investigated through a genome-wide CRISPR knockout screen in A549 cells, a human lung adenocarcinoma line. We identified and validated a variety of novel host factors that play a functional role in LCMV infection. Among these, knockout of the sialomucin CD164, a heavily glycosylated transmembrane protein, was found to ablate infection with multiple LCMV strains but not other hemorrhagic mammarenaviruses in several cell types. Further characterization revealed a dependency of LCMV entry on the cysteine-rich domain of CD164, including an N-linked glycosylation site at residue 104 in that region. Given the documented role of LCMV with respect to transplacental human infections, CD164 expression was investigated in human placental tissue and placental cell lines. CD164 was found to be highly expressed in the cytotrophoblast cells, an initial contact site for pathogens within the placenta, and LCMV infection in placental cells was effectively blocked using a monoclonal antibody specific to the cysteine-rich domain of CD164. Together, this study identifies novel factors associated with LCMV infection of human tissues and highlights the importance of CD164, a sialomucin that previously had not been associated with viral infection.

## INTRODUCTION

The *Arenaviridae* family is classified into four genera: *Antennavirus*, discovered in actinopterygian fish; *Reptarenavirus* and *Hartmanivirus*, which infect boid snakes; and *Mammarenavirus*, whose hosts are predominantly rodents (1–4). *Mammarenavirus* can be further divided into two major virus subgroups based on antigenic properties: Old World (OW), which are mainly indigenous to Africa, and New World (NW), which are indigenous to the Americas (5). Several viruses from this genus can also infect humans, leading to severe or fatal disease. One such pathogenic mammarenavirus is lymphocytic choriomeningitis virus (LCMV). Considered to be the prototypic arenavirus, LCMV is an OW virus found on all populated continents due to the ubiquitous distribution of its natural host, the house mouse (Mus musculus) (6). The prevalence, however, among humans as measured through serological presence of LCMV antibodies widely varies (from 4% to 13%), making it challenging to estimate disease burden and infection risk (7). In addition to contact with infected rodents, humans can also become infected with LCMV through solid-organ transplant or by vertical transmission. In the former case, LCMV infection in immunosuppressed organ recipients is frequently fatal, and the only available therapeutic is off-brand use of the nucleoside analog ribavirin (8). As for the latter, transplacental infection leading to congenital LCMV is typically abortive or results in severe and often fatal fetopathy (9, 10).

Like all other mammarenaviruses, LCMV is a pleiomorphic enveloped virus with a bisegmented, ambisense, negative-stranded RNA genome encoding four genes (11). The L segment (7.2 kb) encodes the viral RNA-dependent RNA polymerase (L) and a small RING finger protein (Z) that is functionally equivalent to the matrix protein found in many enveloped RNA viruses. The S segment (3.4 kb) encodes the viral nucleoprotein (NP) and the glycoprotein complex (GPC). The GPC is synthesized in the infected cell as a precursor polypeptide before being proteolytically processed into a stable signal peptide (SSP) and two noncovalently linked subunits, GP1 and GP2, by the protease SKI-1/S1P (12). GP1 subunit associates with a cellular receptor while GP2 is a transmembrane protein that mediates the pH-dependent fusion of viral and cellular membranes in the late-stage endosomes (13–15). All three subunits remain associated in a tripartite complex while expressed on the viral surface to facilitate viral attachment and entry (12, 16).

Dystroglycan (DAG1), a widely expressed cell adhesion molecule, is recognized as the main attachment factor for viral entry by LCMV, Lassa virus (LASV), and several other NW mammarenaviruses (17, 18). DAG1 is expressed as a precursor polypeptide that is posttranslationally cleaved into two noncovalently associated subunits, the peripheral membrane alpha subunit (αDG) and the transmembrane beta subunit (βDG) (19). Additionally, αDG undergoes complex O-glycosylation mediated by the glycotransferase-like acetylglucosaminyl-transferase (LARGE). Appropriate LARGE-dependent glycosylation is critical for interaction between αDG and mammarenavirus GP (20, 21).

LCMV cellular tropism, however, does not always correlate with the presence of fully glycosylated αDG, and certain strains of LCMV are still found to efficiently bind and infect host cells in the complete absence of DAG1 (22). Previous studies have shown that single amino acid substitutions such as S153F, Y155H, and L260F in the GP1 domain can alter the binding affinity to αDG and shift GP binding preference to alternative receptors (23). This allowed for further classification of LCMV strains into high- and low-αDG LCMV variants. Several secondary receptors have been proposed, including members of the Tyro3/Axl/Mer (TAM) family and heparan sulfate proteoglycans (24–26). Interestingly, in each case, residual viral infection is still observed when tested in genetic knockouts, implying the presence of additional receptors able to mediate cell entry.

The cell entry process reaches completion for mammarenaviruses when viral and cell membrane fusion allows the viral RNP to be deposited into the cytoplasm. For OW mammarenaviruses Lassa virus and Lujo virus, this step requires GP2 to bind to late endosomal resident proteins LAMP1 and CD36, respectively, in a low-pH environment (27, 28). Whether LCMV also requires such a receptor switch in the late endosome is currently unknown.

Although LCMV is considered the prototypic mammarenavirus and is consistently used as a model to study the effect of viral persistence on host immunity, several aspects of its viral life cycle and cellular tropism remain incompletely understood. In this study, we explored the essential host requirements for LCMV infection by performing a genome-wide CRISPR Cas9 knockout (KO) screen using the GeCKOv2 guide library (29). Our results identify new host factors associated with LCMV infection while also corroborating previously implicated factors. Among these results, we identify CD164 as an essential entry factor and possible therapeutic target for LCMV infection.

## RESULTS

### CRISPR KO screens identify host factors for LCMV infection.

LCMV is a virus with minimal cytopathic effect. To conduct a genome-wide pooled CRISPR KO screen to identify host factors important for LCMV infection, a recombinant trisegmented LCMV reporter virus (rLCMV-mCherry) with one L segment and two S segments was constructed (30). We genetically encoded mCherry in place of the nucleoprotein (NP) on one S segment and in place of the glycoprotein complex (GPC) on the other S segment (see Fig. S1A in the supplemental material). One-step growth curves demonstrated slower growth kinetics for rLCMV-mCherry than its parental strain, LCMV Armstrong 53b (Arm 53b), with final titers being comparable (Fig. S1B). Twenty-four hours postinfection (hpi), the percentage of cells expressing mCherry (94.2% mCherry^+^) was equivalent to the percent expressing nucleoprotein (99.6% N protein^+^), suggesting minimal deleterious effects of this trisegmented genome arrangement (Fig. S1C).

10.1128/mbio.00205-22.1 FIG S1 Validation of recombinant virus rLCMV-mCherry infectivity. (A) Schematic representation of LCMV Arm 53b and rLCMV-mCherry genomes. (B) One-step growth curves of wild-type LCMV Arm 53b strain (black) and rLCMV-mCherry made in Arm 53b background (red) as measured by TCID_50_ over a 24-h time course. Error bars indicate standard errors from three independent experiments. (C) Infection percentage of A549 cells infected at multiplicity of infection (MOI) of 0.1, 1, and 10 with wild-type LCMV Arm 53b (top) or r3LCMV-mCherry (bottom) as measured at 24 h postinfection (hpi) using flow cytometry. Cells infected with Arm 53b were stained with anti-LCMV-NP monoclonal antibody (MAb) 113 primary and Alexa 488 secondary and measured for FITC signal compared with an uninfected control. Cells infected with rLCMV-mCherry were measured for PE-CF594 (mCherry) signal compared with an uninfected control. Download FIG S1, TIF file, 0.5 MB. Copyright © 2022 Liu et al. 2022 Liu et al. https://creativecommons.org/licenses/by/4.0/ This content is distributed under the terms of the Creative Commons Attribution 4.0 International license.

**FIG 1:**
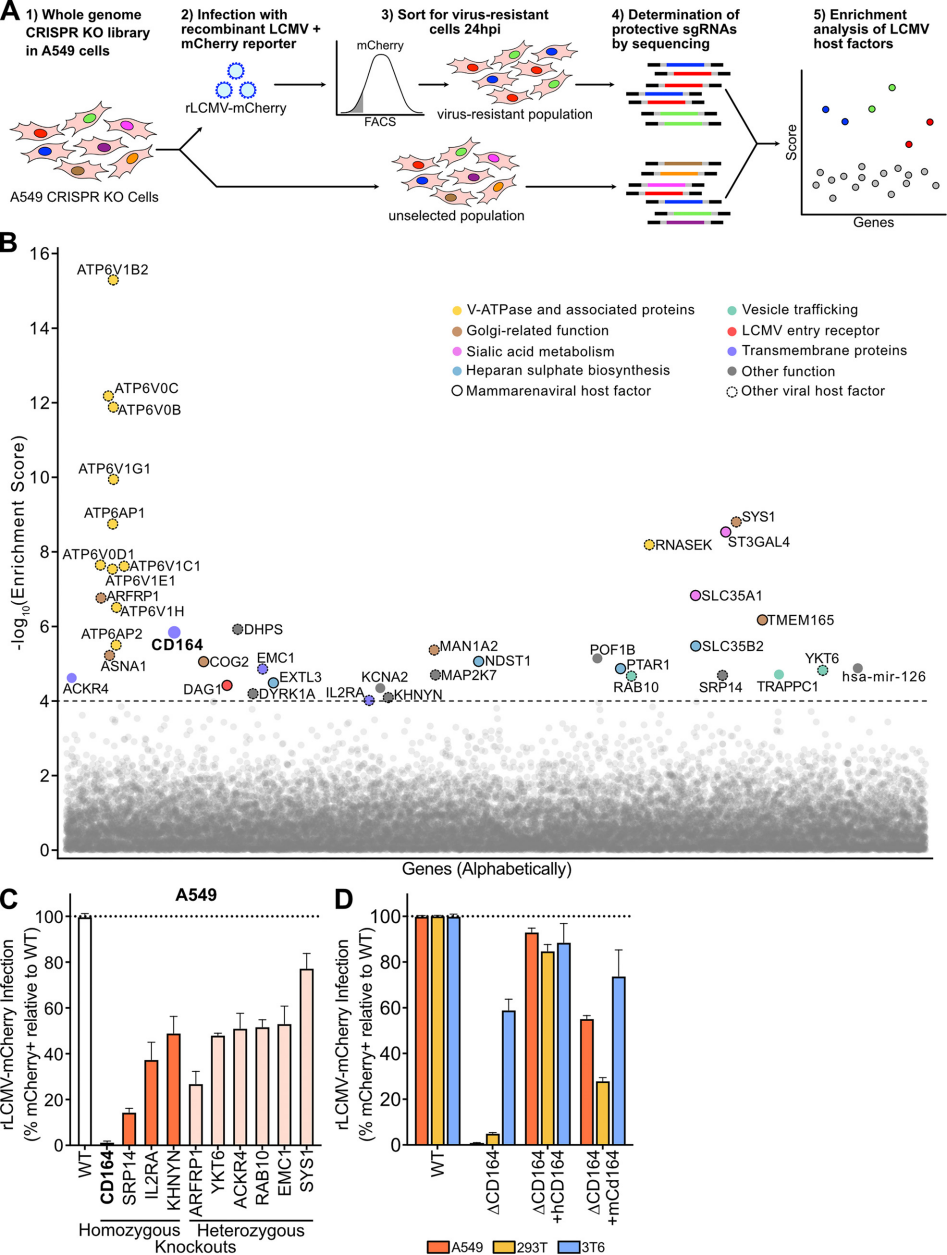
Genome-wide CRISPR loss-of-function screen in human cells identify host factors important for LCMV infection. (A) Schematic of CRISPR-based KO screen done in A549 lung epithelial cells for the identification of LCMV host factors. (B) Gene enrichment for CRISPR screen of rLCMV-mCherry infection. Enrichment scores were determined by MaGECK analysis, and genes were colored by biological function. Dotted line indicates −log_10_(enrichment score) = 4. All genes and their enrichment scores can be found in Table S1. (C) Percentage of infected cells as determined by flow cytometry following infection of A549 homozygous knockouts (CD164, SPR14, IL2RA, and KHNYN) or heterozygous knockouts (ARFRP1, YKT6, ACKR4, RAB10, EMC1, and SYS1) with rLCMV-mCherry. Wild-type cells were used as normalization controls. Cells were infected at an MOI of 1 and harvested at 24 hpi. Error bars indicate standard errors from three independent experiments. (D) Quantification of viral infection in WT, Δ*CD164*, Δ*CD164* complemented with human *CD164* (Δ*CD164 *+* hCD164*), and Δ*CD164* complemented with mouse *Cd164* (Δ*CD164 *+* mCd164*) in A549, 293T, and 3T6 cell type backgrounds. Cells were infected with rLCMV-mCherry at an MOI of 1 and harvested at 24 hpi. Error bars indicate standard errors from three independent experiments.

As inhalation of aerosolized virus is a major transmission route, human adenocarcinoma lung epithelial cells (A549) were the chosen cell line for whole-genome CRISPR KO screening with the GeCKOv2 guide library. Following rLCMV-mCherry infection (multiplicity of infection [MOI] of 10) of the A549 CRISPR KO library cells, mCherry-negative cells were sorted 24 hpi to select for single-guide RNAs (sgRNAs) targeting host factors necessary for successful LCMV infection (Fig. 1A). The sgRNAs present in this virus-resistant population and an unsorted control population were PCR amplified from the extracted genomic DNA and subsequently identified via next-generation sequencing. Using the MAGeCK algorithm, genes were ranked using robust rank aggregation to produce a significance score called the MAGeCK enrichment score (31). As expected, multiple sgRNAs targeting the same gene were among the top scoring guides, including those targeting previously described mammarenavirus host factors (Fig. 1B and Fig. S2A, Table S1). These include sialic acid metabolism genes (*ST3GAL4* and *SLC35A1*) and glycosylation-related genes (conserved oligomeric Golgi [COG] complex members, *TMEM165*), which have been shown to be LASV host factors (32). Multiple heparan sulfate biosynthetic genes (*EXTL3*, *NDST1*, *PTAR1*, and *SLC35B2*) described to be relevant for Lujo virus and DAG1-independent LCMV infections were also enriched (25, 28). The LCMV attachment factor *DAG1* was detected, albeit at a lower enrichment score. Additional host factors that were significantly enriched include those described for other viral infections, such as negative-stranded RNA virus vesicular stomatitis virus (VSV) (*ARFRP1*, *SYS1*, and *YKT6*) and the human immunodeficiency virus (HIV) (*SRP14*, *DYRK1A*, and *IL2RA*) (33–37).

10.1128/mbio.00205-22.2 FIG S2 Additional hit validation and characterization of gene-edited cells. (A) Log fold changes (LFC) of individual sgRNA of the top 10 scoring genes and CD164 (red) when comparing the infected and sorted cell population versus the uninfected cell population. Overall sgRNA distribution is shown at the bottom of the graph, and dotted line indicates mean LFC of all sgRNAs. (B to D) Dose-response curve of v-ATPase inhibitors on rLCMV-mCherry infection at MOI of 1 in A549 cells at 24 hpi, yielding Bafilomycin A_1_ IC_50_ = 2.96 nM (B), Bafilomycin B_1_ IC_50_ = 4.97 nM (C), and concanamycin A IC_50_ = 0.83 nM (D). Error bars indicate standard errors from three independent experiments. (E) Genotyping of clonal A549 where the target loci were PCR amplified, Sanger sequenced, and aligned to WT reference sequence. (F) Analysis of cell proliferation of WT and clonal A549 KO cells. Cells were plated in 96 wells and proliferation was measured daily using Cell Titer Glo. Error bars indicate standard error from three separate well per cell line per time point. (G to I) Western blot analysis of WT, Δ*CD164*, Δ*CD164 *+* hCD164*, and Δ*CD164 *+* mCd164* for A549, 293T, and 3T6 cell lines. Human cell lines (A549 and 293T) were probed with anti-hCD164 antibody except for the *mCd164* addback, which was probed with anti-mCd164 antibody. Mouse cell line 3T6 was probed with anti-mCd164 antibody except for the *hCD164* addback, which was probed with anti-hCD164 antibody. GAPDH was used as a loading control. (J) One-step growth curves of rLCMV-Cherry on A549 WT or A549 ΔCD164 cells as measured by TCID_50_ over a 72-h time course. Error bars indicate standard errors from two independent experiments. Download FIG S2, TIF file, 2.3 MB. Copyright © 2022 Liu et al. 2022 Liu et al. https://creativecommons.org/licenses/by/4.0/ This content is distributed under the terms of the Creative Commons Attribution 4.0 International license.

10.1128/mbio.00205-22.7 TABLE S1 rLCMV-mCherry whole-genome CRISPR screen results. Table of the top genes identified by the rLCMV-mCherry whole-genome CRISPR screen, along with the MAGeCK enrichment scores, *P* values, false discovery rate, rank position, number of relevant sgRNAs, log fold change, and each ranking position of the gene’s respective sgRNAs. Download Table S1, XLSX file, 0.04 MB. Copyright © 2022 Liu et al. 2022 Liu et al. https://creativecommons.org/licenses/by/4.0/ This content is distributed under the terms of the Creative Commons Attribution 4.0 International license.

Gene Ontology (GO) overrepresentation analysis of the top 300 hits from the screen using PANTHER (38) indicated an enrichment of genes associated with the signal recognition particle (*SRP14*, *SRP68*, *SRP19*, and *SRP72*) and proton transmembrane transporter activity (*ATP6V1E1*, *ATP6V0D1*, *ATP6V1B2*, *ATP6V0C*, *ATP6V1A*, *ATP6V1G1*, *ATP6V0B*, *ATP12A*, *ATP6V1H*, *ATP6V1C1*, *CLCN4*, *ATP6V1F*, and *ATP5S*) (Table S2). These same hits are also overrepresented in GO cellular component signal recognition particles and vacuolar proton-transporting vacuolar type ATPase (v-ATPase) complex, respectively.

10.1128/mbio.00205-22.8 TABLE S2 Gene ontology overrepresentation analysis. Results of using the PANTHER Overrepresentation test to analyze the top hits identified through the rLCMV-mCherry whole-genome CRISPR screen. Fold enrichment, raw *P* value, false discovery rate, as well as enriched gene members were identified for the gene ontologies biological process, molecular function, and cellular component. Download Table S2, XLSX file, 0.01 MB. Copyright © 2022 Liu et al. 2022 Liu et al. https://creativecommons.org/licenses/by/4.0/ This content is distributed under the terms of the Creative Commons Attribution 4.0 International license.

Nearly every subunit of the v-ATPase (*ATP6V1B2*, *ATP6V0C*, *ATP6V0B*, *ATP6V1G1*, *ATP6AP1*, *ATP6V0D1*, *ATP6V1C1*, *ATP6V1E1*, *ATP6V1H*, and *ATP6AP2*) was enriched in our screen. v-ATPase is a proton pump responsible for acidification of intracellular systems, a process necessary for the required pH-dependent fusion event between LCMV viral and cellular membranes in the acidic environment of the late-stage endosome (15, 39). To validate this screening result, known v-ATPase inhibitors bafilomycin A_1_ (Fig. S2B), bafilomycin B_1_ (Fig. S2C), and concanamycin A (Fig. S2D) were tested for efficacy in LCMV infection inhibition (40). As expected, all three drugs exhibited dose-dependent protection against LCMV infection in A549 cells with nanomolar efficacy, consistent with the critical role v-ATPase plays in LCMV infection.

To explore other candidate genes of interest identified in this screen, monoclonal A549 knockout (KO) cell lines containing frameshift mutations were generated for several top-scoring genes (Fig. S2E). These cells lines were also tested for normal cell growth (Fig. S2F). Among these candidates were the transmembrane proteins encoded by *ACKR4*, *CD164*, *EMC1*, and *IL2RA*; the *trans*-Golgi/endosome membrane trafficking complex genes *ARFRP1* and *SYS1*; the vesicular transport-associated genes *YKT6* and *RAB10*; the ZAP antiviral protein cofactor *KHNYN*; and the signal recognition particle gene *SRP14.* In all cases, homozygous and heterozygous knockouts in A549 cells yielded significant decreases in LCMV infection, ranging from severely impaired relative to wild type (WT), 1.3% infected (*CD164*^−/−^), to moderately impaired, 77% infected (*SYS1*^−/+^) (Fig. 1C).

Since knockout of CD164 demonstrated near ablation of infection, we chose to follow up on this protein to explore its role in the viral life cycle. CD164 is a heavily glycosylated transmembrane sialomucin cell adhesion protein expressed in a wide range of tissues (41, 42). This gene was originally characterized as a marker for CD34^+^ hematopoietic progenitor cells, where it may be involved in a variety of processes, including cellular adhesion, autophagy, tumorigenesis, and metastasis (43, 44). To date, *CD164* has not been associated with any known viral entry mechanisms.

To further investigate the role of this gene in LCMV infection, monoclonal *CD164* KO (Δ*CD164*) cell lines were generated in two additional cell types, 293T (human embryonic kidney cells) and 3T6-Swiss albino (mouse embryonic fibroblast cells). In both human lines, A549 and 293T, deletion of CD164 reduced infection by 99% and 95%, respectively, while the effect in the mouse cell line 3T6 was moderate (41% reduction) (Fig. 1D). Infectivity of each KO cell line was nearly fully restored by complementation with ectopically expressed human *CD164* (*hCD164*) gene driven by the LCMV promoter. Complementation with the mouse *Cd164* (*mCD164*) gene, which is 62.32% identical on a protein level, partially restored infectivity in all three cell lines. We confirmed protein expression levels in knockout and complemented cell lines by Western blotting (Fig. S2G to I).

In addition to viral infections, one-step growth curves were also conducted to determine the abundance of infectious viral progeny produced by *ΔCD164* cells (Fig. S2J). As expected, few productive virions were produced from *ΔCD164* A549 cells compared to the WT A549 cells. Together, our data suggest that *CD164* is essential for LCMV infection in human cells.

### Pseudotyped viral infection shows that CD164 is an LCMV-specific mammarenavirus human entry factor.

Previous work has demonstrated *DAG1* to be an entry-related attachment receptor in mice (17). Our screen also identified DAG1 as an important LCMV entry factor in addition to implicating CD164 as a determinant of human cell entry for LCMV. To further explore the dependency of mammarenaviruses on *CD164* or *DAG1* for viral entry, we generated and validated additional monoclonal KO cell lines, Δ*DAG1* and Δ*CD164* Δ*DAG1* double KO lines, in both A549 (Fig. S3A) and 293T (Fig. S3B) cell backgrounds. To specifically test the entry stage of the viral life cycle, recombinant green fluorescent protein (GFP) expressing vesicular stomatitis virus [rVSV-ΔG(GFP)] pseudotyped with a panel of mammarenavirus GP were utilized (45). The advantage of this method is twofold: targeted examination of GP receptor tropism in the absence of other factors that may influence native viral infection and the ability to study biosafety level 4 (BSL-4) pathogenic mammarenaviruses under standard BSL-2 laboratory conditions (46).

10.1128/mbio.00205-22.3 FIG S3 Characterization of Δ*CD164*, Δ*DAG1*, and Δ*CD164*/Δ*DAG1* double KO cells. (A and B) Western blot analysis of Δ*CD164*, Δ*DAG1*, and Δ*CD164*/Δ*DAG1* double KO cells in A549 (A) or 293T (B) cell backgrounds. GAPDH was used as a loading control. Download FIG S3, TIF file, 0.2 MB. Copyright © 2022 Liu et al. 2022 Liu et al. https://creativecommons.org/licenses/by/4.0/ This content is distributed under the terms of the Creative Commons Attribution 4.0 International license.

**FIG 2:**
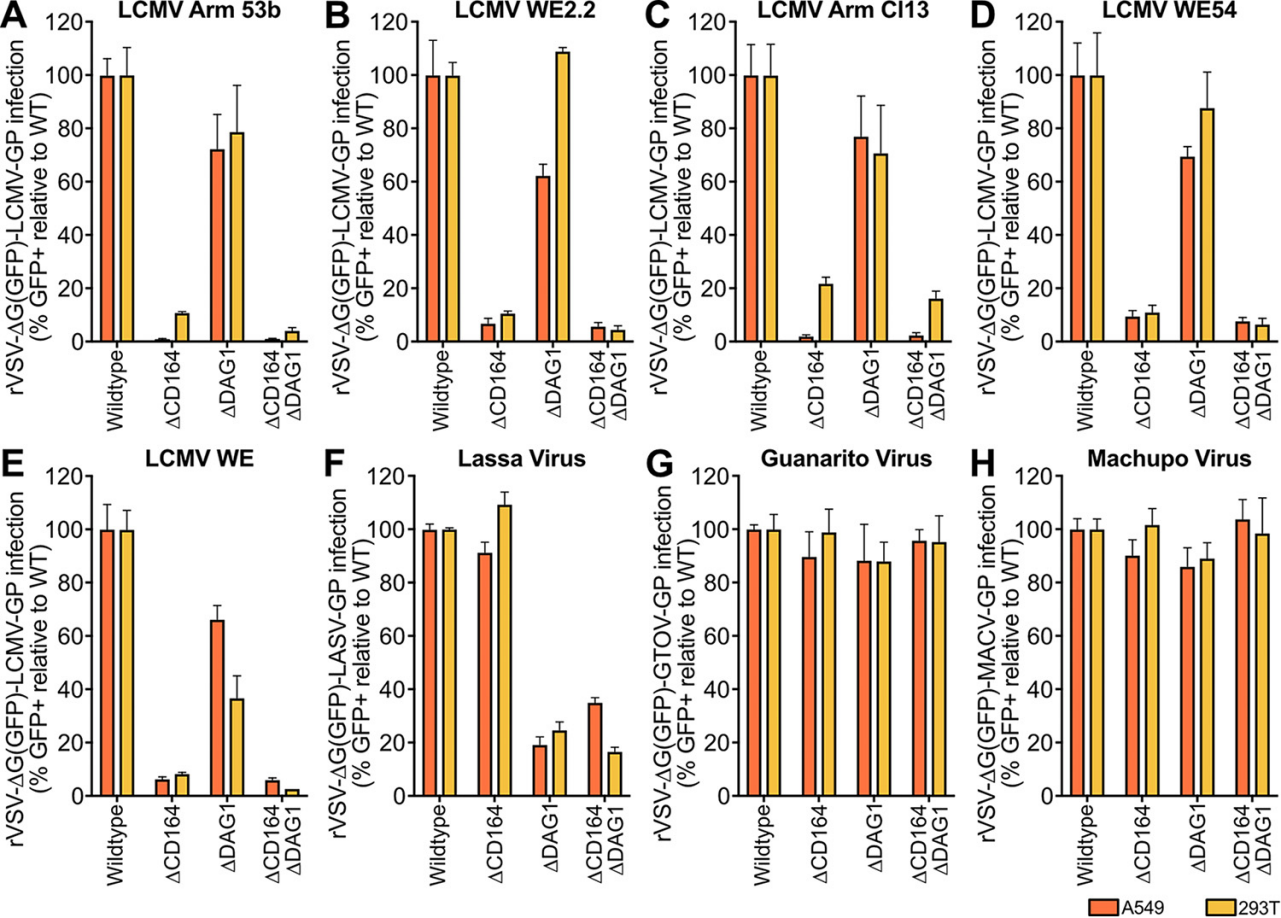
Infection of KO cell lines with a panel of mammarenavirus GP pseudotyped virus. (A to E) Percent infection of Δ*CD164*, Δ*DAG1*, and Δ*CD164* Δ*DAG1* double-KO cells relative to WT in either A549 or 293T cell type backgrounds following inoculation with low-DAG1-affinity LCMV strain Armstrong 53b-GP (A) or WE2.2-GP (B) and high-DAG1-affinity strain Armstrong clone 13-GP (C), W54-GP (D), or WE-GP (E) pseudotyped virus as determined by flow cytometry for GFP positivity. Cells were infected at an MOI of 1 and measured 24 hpi. Error bars indicate standard errors from three independent experiments. (F to H) Percent infection of ΔCD164, ΔDAG1, and ΔCD164 ΔDAG1 double-KO cells relative to WT in either A549 or 293T cell type backgrounds following inoculation with LASV-GP (D), GTOV-GP (E), or MACV-GP (F) pseudotyped virus as determined by flow cytometry for GFP positivity. Cells were infected at an MOI of 1 and measured 24 hpi. Error bars indicate standard errors from three independent experiments.

The GPs from several LCMV strains representing a range of DAG1 affinities were combined with rVSV-ΔG(GFP) to generate pseudotyped virus (Fig. 2A to E). Arm 53b (used for the CRISPR screen) (Fig. 2A) and WE2.2 (Fig. 2B) represent low-DAG1-affinity strains, while Armstrong clone 13 (Arm Cl13) (Fig. 2C), WE54 (Fig. 2D), and WE (Fig. 2E) were chosen to represent high-DAG1-affinity strains (22, 23, 25). Deletion of *CD164* reduced infection by all four pseudotyped viruses by 78% to 99% in both human cell lines, indicating a strong CD164 dependency in all cases. In contrast, knockout of *DAG1* in both A549 and 293T cells led to only moderate decreases in pseudotyped virus infection (23% to 38% reduction for A549; up to 63% reduction for 293T) across all LCMV strains. In one case [293T Δ*DAG1* infected with rVSV-ΔG(GFP)+WE54-GP], deletion of *DAG1* had virtually no measurable impact on pseudotyped virus infection. Consistent with that, Δ*DAG1 ΔCD164* double KO cells yielded decreased pseudovirus infection similar to or below those observed in *CD164* KO cells. Together, these results suggest that *CD164* is the major determinant for LCMV entry in both A549 and 293T cells, whereas *DAG1* plays only an accessory role in these human cell types.

To extend these findings beyond LCMV, a selection of hemorrhagic mammarenavirus GPCs were used to generate pseudovirus for infection in A549 and 293T cells. As previously described, *DAG1* is an important entry factor for the OW mammarenavirus Lassa virus (LASV) but not for the NW mammarenaviruses Guanarito virus (GTOV) and Machupo virus (MACV) (17, 18). Consistent with these findings, deletion of *DAG1* abrogated LASV entry (Fig. 2F) but had minimal effects on GTOV (Fig. 2G) and MACV (Fig. 2H) entry. In contrast, deletion of *CD164* had no effect on infection for any of the three tested pathogenic mammarenaviruses, suggesting that *CD164* is a critical human entry factor for LCMV but not other mammarenaviruses. Using GP-pseudotyped virus, we have determined that *CD164* plays a major functional role for LCMV entry in human cells and no other tested hemorrhagic mammarenaviruses, while *DAG1* is an important entry factor for LASV and, to a lesser extent, certain strains of LCMV.

### N-linked glycosylation within the cysteine-rich domain is critical for LCMV infection.

*CD164* is a 197-amino-acid type 1 integral transmembrane protein featuring a 14-amino-acid intracellular tail and a 139-amino-acid extracellular region that is expressed as a homodimer nearly ubiquitously throughout human tissues (47). The extracellular portion of CD164 is comprised of two mucin domains flanking a cysteine-rich domain. The protein also features one predicted attachment site for O-linked glycans and 9 predicted N-linked glycosylation sites throughout the mucin- and cysteine-rich domains.

**FIG 3:**
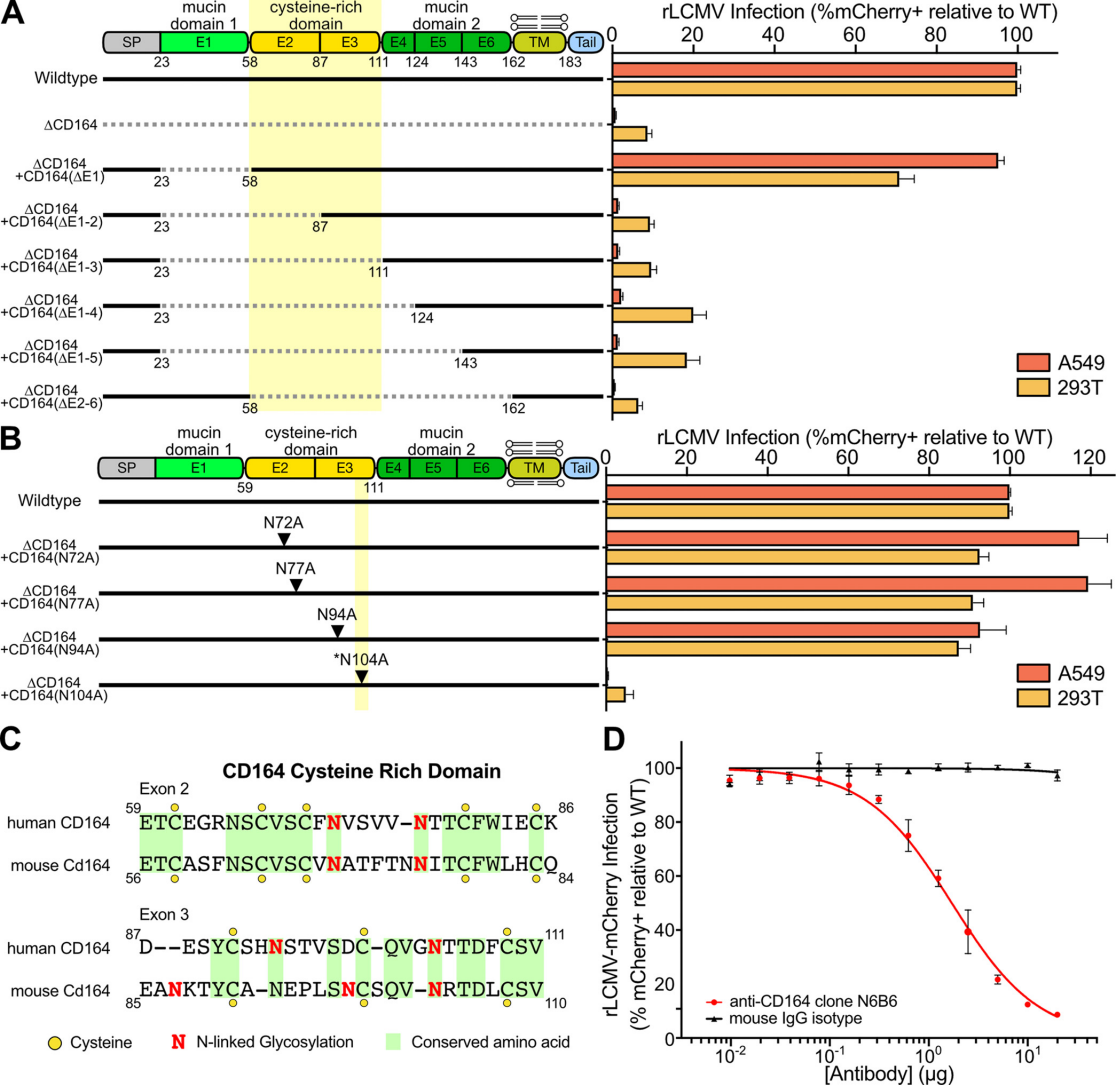
CD164 functional region determination through antibody binding, domain deletion, and alanine mutagenesis. (A, left) Schematic of wild-type, Δ*CD164*, Δ*CD164 *+* hCD164*(ΔE1), Δ*CD164 *+* hCD164*(ΔE1-2), Δ*CD164 *+* hCD164*(ΔE1-3), Δ*CD164 *+* hCD164*(ΔE1-4), Δ*CD164 *+* hCD164*(ΔE1-5), and Δ*CD164 *+* hCD164*(ΔE2-6). (Right) Complemented A549 and 293T cells were challenged with rLCMV-mCherry (MOI, 1) and infection was measured by flow cytometry at 24 hpi. Percent infection was normalized to wild type. Error bars represent standard errors from three independent experiments. (B, left) Schematic of wild type, ΔCD164 KO + hCD164(N72A), ΔCD164 + hCD164(N77A), ΔCD164 + hCD164(N94A), and ΔCD164 + hCD164(N104A). (Right) Complemented A549 and 293T cells were challenged with rLCMV-mCherry (MOI, 1) and infection was measured by flow cytometry at 24 hpi. Percent infection was normalized to the wild type. Error bars represent standard errors from three independent experiments. (C) Amino acid similarities of the cysteine-rich region in human CD164 and mouse Cd164 determined using the ClustalW program on SnapGene. Yellow circles indicate cysteine residues, red N symbolizes N-linked glycosylation sites, and identical amino acids are highlighted in green. (D) Blockade of LCMV infection with serial dilutions of anti-human CD164 monoclonal mouse antibody clone N6B6 or mouse IgG2a-κ isotope control in wild-type A549 cells. Cells were infected at an MOI of 1 and infection measured at 24 hpi. Error bars indicate standard errors from three independent experiments.

To further dissect the role of *CD164* with respect to LCMV entry, a series of *CD164* domain deletion mutants were constructed and introduced into A549 Δ*CD164* and 293T Δ*CD164* cells (Fig. 3A). Deletion of the first mucin domain [Δ*CD164 *+* hCD164*(ΔE1)] did not affect infection, suggesting this domain is not necessary for LCMV entry. Extending the deletion into the cysteine-rich domain [Δ*CD164 *+* hCD164*(ΔE1-2)], however, ablated infection (mean 98% reduction for A549; 86% reduction for 293T), thereby phenocopying the ΔCD164 cells. We confirmed expression of all domain deletion constructs by Western blotting (Fig. S4A and B).

10.1128/mbio.00205-22.4 FIG S4 Characterization of CD164 domain deletion and alanine mutagenesis add backs. (A and B) Western blot analysis of deletion domain addbacks in (A) A549 or (B) 293T cell backgrounds. (C and D) Western blot analysis of alanine mutagenesis addbacks in (C) A549 or (D) 293T cell backgrounds. All CD164 addbacks were probed with anti-FLAG antibody. GAPDH was used as a loading control. (E) AlphaFold prediction of CD164 protein structure. Prediction had low position error for the signal peptide, the cysteine-rich region, the transmembrane domain, and the cytoplasmic tail and high position error for the two mucin domains. Location of residue 104 is noted with an arrow. Download FIG S4, TIF file, 1.5 MB. Copyright © 2022 Liu et al. 2022 Liu et al. https://creativecommons.org/licenses/by/4.0/ This content is distributed under the terms of the Creative Commons Attribution 4.0 International license.

The cysteine-rich region of *CD164* contains four putative N-linked glycosylation sites (Fig. 3B). To test the importance of these sites individually, alanine substitutions were introduced in place of each relevant asparagine, and expression of these mutant constructs was confirmed by Western blotting (Fig. S4C and D). Mutation of N-linked glycosylation sites at positions 72, 77, and 94 did not reduce infection by rLCMV-mCherry; however, substitution of N104 completely abolished infection. This asparagine residue, which is conserved between human and mouse CD164 (Fig. 3C), sits in a loop region between a beta-sheet and an alpha-helix as predicted by AlphaFold (Fig. S4E) (48). The ablation of infection due to mutagenesis of the N-linked glycosylation site suggests that the cysteine-rich domain, including a critical asparagine amino acid, is required for *CD164*-mediated infection by LCMV.

The deletion mapping of *CD164* indicated the importance of the cysteine-rich domain. To further explore this domain, we tested whether an anti-CD164 monoclonal antibody (MAb) could competitively inhibit LCMV infection. The anti-CD164 MAb N6B6, which was demonstrated to bind a conformationally dependent backbone epitope encompassing the cysteine-rich domain between the two mucin domains (42, 49), blocked infection by rLCMV-mCherry in a dose-dependent manner (Fig. 3D). These results are consistent with the deletion mapping and alanine mutagenesis data, highlighting the importance of the central cysteine-rich domain for LCMV infection.

### CD164 is highly expressed in human placenta and mediates LCMV infection in placental cells.

Although LCMV infection as a child or an adult is typically inconsequential, infection during pregnancy can lead to transplacental human fetal infections with severe clinical consequences (10). Like many other congenital pathogens, LCMV has tropism for fetal neural and retinal tissue, leading to developmental issues such as microencephaly, macrocephaly, chorioretinitis, periventrictular calcification, and hydrocephalus (50, 51). Retrospective studies on serologically confirmed cases show that children with congenital LCMV infection have a 35% mortality rate by 2 years of age and survivors experience long-term neurological, motor, and visual impairments (9, 52).

**FIG 4:**
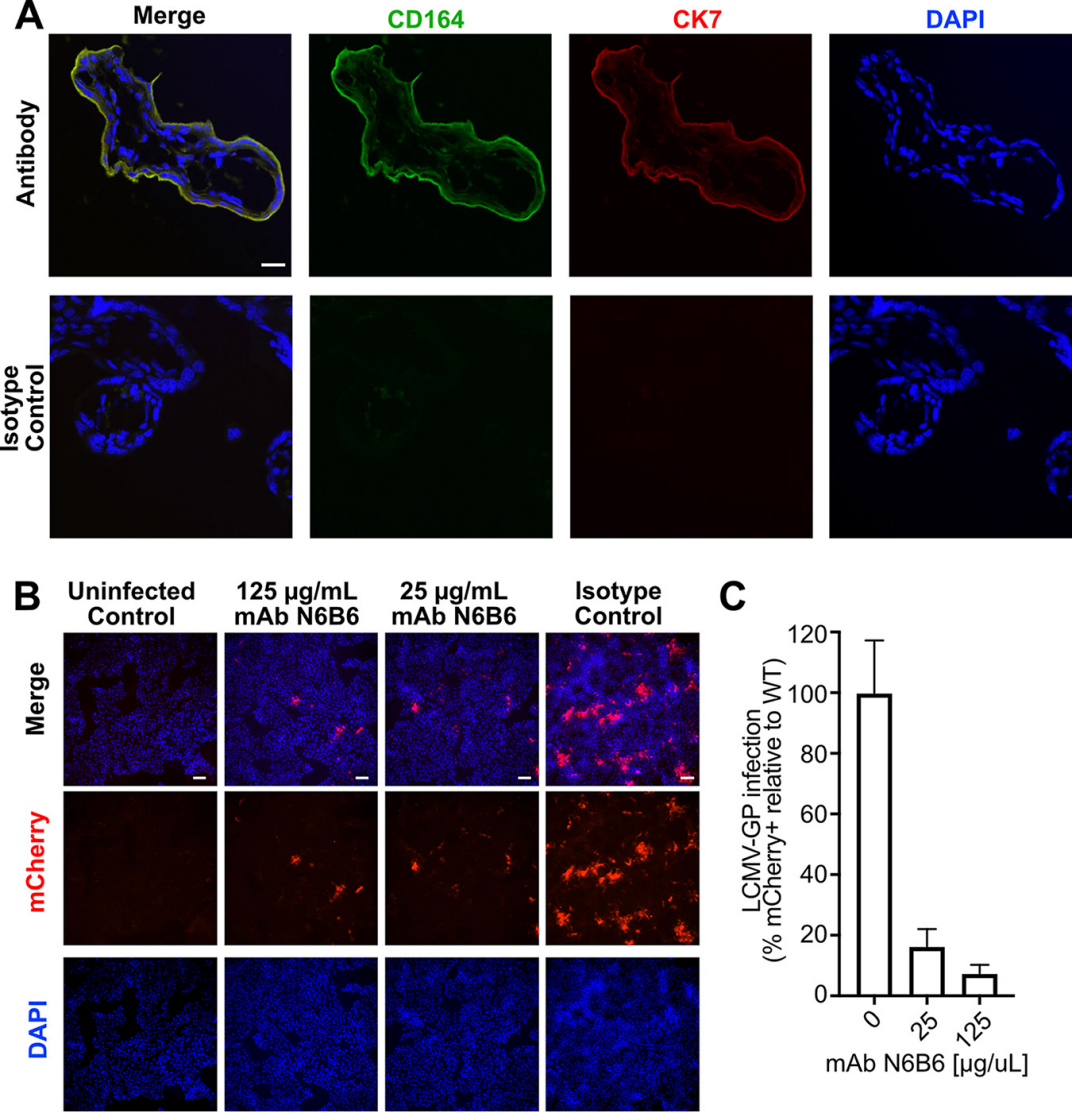
Characterization of CD164 as a therapeutic target in human placenta. (A) Double immunofluorescence staining for CD164 and CK7 or isotype staining followed by counterstaining with DAPI in villous trophoblastic tissue. Original images were taken by confocal microscopy at ×100 magnification. Scale bar represents 20 μm. (B) Immunofluorescence imaging of JEG-3 placenta cells preincubated with various concentrations of anti-CD164 MAb N6B6 and infected with r3LCMV-mCherry at an MOI of 0.5. Cells were fixed and imaged at ×10 magnification 24 hpi. Scale bar represents 20 μm. (C) Quantification of percent infection of JEG-3 placenta cells preincubated with various concentrations of anti-CD164 MAb N6B6 and infected with r3LCMV-mCherry at MOI of 0.5. Analysis was done on 4 fields of view in 2 independent infections and normalized to infection control.

Human fetal vulnerability to LCMV led us to hypothesize that CD164 plays a role in transplacental infection. To explore tissue-specific expression of CD164 during pregnancy, healthy second trimester placentas were costained with CD164 MAb anti-CD164 N6B6. CD164 was highly expressed in the outer layer of floating chorionic villi and absent from the underlying mesenchyme; colocalization with cytokeratin-7 confirmed that CD164 was expressed in cytotrophoblasts (Fig. 4A) (53). In contrast, CD164 was not detected in the decidua (maternal side), pointing to a fetus-specific localization at this interface (Fig. S5A). Cytotrophoblasts bathe in maternal blood and are an initial contact site for pathogens (54), suggesting that CD164 is present in tissue structures and locations amenable for transplacental infection of the developing fetus.

10.1128/mbio.00205-22.5 FIG S5 Characterization of CD164 in placenta tissue and cell line. (A) Immunofluorescence staining of CD164 or isotype control followed by counterstaining with DAPI on placenta tissue at the maternal decidua and fetal villi. Original images taken at 40× magnification. Scale bar represents 50 μm. (B) Immunofluorescence imaging of CD164 or isotype control followed by counterstaining with DAPI on JEG-3 placenta cell line. Original images taken at 10× magnification. Scale bar represents 100 μm. (C) Immunofluorescence imaging JEG-3 placenta cell line with and without infection by r3LCMV-mCherry at MOI of 1 and imaged at 24 hpi. Original images taken at 10× magnification. Scale bar represents 100 μm. Download FIG S5, TIF file, 3.0 MB. Copyright © 2022 Liu et al. 2022 Liu et al. https://creativecommons.org/licenses/by/4.0/ This content is distributed under the terms of the Creative Commons Attribution 4.0 International license.

CD164 expression was also observed in a JEG-3 human chorionic cell line, which we have demonstrated to be permissive to LCMV infection (Fig. S5B and C). Preincubation of these cells with anti-CD164 MAb N6B6 blocked rLCMV-mCherry infection, with a treatment of 25 μg/mL N6B6 reducing the detection of mCherry-positive cells by 84% and 125 μg/mL reducing infection further by 94% (Fig. 4B and C). This dose-dependent inhibition of LCMV infection indicates that LCMV utilizes CD164 ectodomains in placental cells. Thus, blocking CD164-mediated entry with a targeted antibody may be a viable therapeutic intervention for congenital LCMV.

## DISCUSSION

In this study, we performed a genome-wide CRISPR KO screen to identify host factors important for LCMV. By using a reporter virus, our screen is well suited to specifically identify host factors involved in viral entry and viral protein production. Furthermore, we chose to conduct our screen using a high MOI to decrease the probability of enriching sgRNAs that may be present in our sorted uninfected population due to chance. Our results ultimately highlight a subset of genes that appear to be shared generally among mammarenaviruses. These genes span pathways and functions such as sialic acid metabolism, heparan sulfate biosynthesis, glycosylation and Golgi trafficking, and late-stage endosome acidification (25, 28, 32). Most notably, we identified 10/24 of the v-ATPase subunits (5 in each of the V_1_ and V_0_ domains) as well as four signal recognition particle subunits. The previously described LCMV entry receptor, *DAG1*, was moderately enriched, consistent with the use of the low-DAG1-affinity Arm 53b LCMV strain in this screen (22, 23).

This screen also revealed genes, most notably CD164, that previously have not been linked to LCMV infection, perhaps facilitated by using a human epithelial lung cell line. We found that in the absence of CD164 in human cells, LCMV infection is nearly ablated. In contrast, mouse Δ*CD164* cells yielded only moderate reduction in LCMV infection. Consistent with this, when complemented with ectopically expressed human or mouse CD164, human CD164 restored infectivity, while the mouse homologue of CD164, which is 62% identical on the protein level, only partially restored infectivity. CD164 localizes to the cell surface and late-stage endosomes, consistent with the LCMV entry route for successful infection (47). Like DAG1, it is a ubiquitously expressed cell adhesion molecule present in nearly all tested human tissue. Unlike DAG1, to which LCMV strains show a range of affinities, all five LCMV strains tested here required CD164 for infection in human cells. Deletion of DAG1 partially reduced infectivity by LCMV, particularly in those previously described as having high DAG1 affinity (Cl13, WE54, and WE). These data strongly support that human lung cells require CD164, and not necessarily DAG1, for viral infection by LCMV, while mouse cells appear to rely on CD164 only partially.

Further characterization of *CD164* by deletion mapping and alanine mutagenesis suggests that the cysteine-rich domain, particularly a single critical N-linked glycosylation site, is required for CD164-mediated infection. The importance of the cysteine-rich domain was reinforced by blocking using the anti-CD164 MAb N6B6, whose presence can inhibit LCMV infection in a dose-dependent manner. These data together suggest that binding by N6B6 to CD164 renders the critical interaction region inaccessible, thus preventing LCMV infection.

While LCMV infection is generally mild among adults and children, clinical outcomes following congenital infections tend to be severe. LCMV transplacental infections are typically fatal, and survivors experience long-term neurological, motor, and visual impairments (9, 10, 52). While off-brand use of ribavirin occurs in cases of LCMV infection following solid-organ transplants, no current treatment procedure exists for congenital LCMV (8). We demonstrated that CD164 localizes to the placental villous cytotrophoblasts, the maternal-fetal interface that is the initial contact site with congenital pathogens. The function of this sialomucin in placentation is unknown, and, to our knowledge, this is the first description of CD164 expression in human placenta. Placental sialoglycoproteins disarm maternal immune recognition of fetal antigens (55), thus making CD164 a particularly elegant target for viral entry. LCMV may evade immunity at this critical interface by coopting natural mechanisms of maternal immune tolerance of her fetus. Once in the fetus, LCMV exhibits brain tropism, specifically targeting neuroblasts. The developing brain is most susceptible as more severe pathology is observed when infection occurs early in gestation, possibly due to incomplete blood-brain barrier formation (51). LCMV also induces fetal inflammation, which is highly damaging and can lead to inflammation-induced cerebral lesions in children with congenital infection (51, 56). Thus, either directly or indirectly, the ability of LCMV to enter the protected fetal compartment, likely via engagement of CD164, contributes to the devastating neurological pathology associated with the virus. Our identification of a key placental entry factor for LCMV and demonstration that an antibody can inhibit this interaction opens the possibility of targeting this pathway for therapeutic intervention.

While this study was under review, a report by Bakkers et al. (57) was published detailing the role of CD164 in relation to LCMV-GP during the viral entry process. Using a LCMV-pseudotyped virus and an infection period of 3 weeks, the authors identified seven host factors important for LCMV entry, four of which (CD164, ST3GAL4, SLC35A1, and MAN1A2) were contained within the set of 37 factors identified in our live virus screen (Fig. S6). CD164 was also demonstrated to be required for low-pH triggered membrane fusion. Similar to LASV, which binds to DAG1 at the cell surface and subsequently uses a receptor switching strategy to transition to LAMP1 binding in the acidic environment of the late-stage endosome (27), LCMV is believed to use a similar mechanism by changing receptors to CD164 for viral membrane fusion.

10.1128/mbio.00205-22.6 FIG S6 Gene enrichment score comparison between two whole-genome CRISPR screens for LCMV host factors. The results of the whole-genome CRISPR screen of this study using the GeCKO v2 sgRNA library and rLCMV-mCherry compared to the results of Bakkers et al. (57) using the Brunello sgRNA library and VSV-LCMV pseudovirus. Enrichment scores are determined by MaGECK analysis. Genes enriched exclusively in this rLCMV-mCherry screen are colored in red. Genes enriched exclusively in the VSV-LCMV screen (SPPL3, MGAT1, and SLC39A9) are colored in blue. Genes enriched in both screens (ST3GAL4, SLC35A1, CD164, and MAN1A2) are colored in purple. All genes and their enrichment scores for the rLCMV-mCherry screen can be found in Table S1. Download FIG S6, TIF file, 0.6 MB. Copyright © 2022 Liu et al. 2022 Liu et al. https://creativecommons.org/licenses/by/4.0/ This content is distributed under the terms of the Creative Commons Attribution 4.0 International license.

The data presented in this study are consistent and complementary to that presented by Bakkers et al. In addition, these data extend identification of host factors important for LCMV infection to 33 additional genes, including members of complexes such as the v-ATPase. Furthermore, we have demonstrated that a monoclonal antibody, N6B6, can bind to CD164 and inhibit LCMV infection in a dose-dependent manner. In addition to lung epithelial cells, this antibody is also shown to be effective in preventing infection in placental cells, thereby presenting a possible therapeutic for preventing congenital LCMV infection.

The demonstration of CD164 as an essential determinant for LCMV entry into human cells and tissue fills an important gap in our understanding of this virus. Whether the reliance on CD164 is unique to LCMV or whether this entry factor is utilized by additional viruses remains unknown. Given the apparent unique dependency of LCMV on CD164 and the practical implications of its involvement in transplacental infection, further exploration of the mechanistic details by which congenital LCMV can be prevented through blocking CD164 is warranted.

## MATERIALS AND METHODS

### Cell lines.

A549, 293T, 3T6, BHK-21, and Vero cells (all from ATCC) were cultured in Dulbecco’s modified Eagle medium (DMEM) (Gibco) supplemented with 10% fetal bovine serum (FBS; Gibco), penicillin-streptomycin-glutamine (Gibco), and HEPES (Gibco) at 37°C and 5% CO_2_. JEG-3 cells (ATCC) were cultured in essential modified Eagle medium (EMEM) (ATCC) supplemented with 10% FBS (Gibco), penicillin-streptomycin-glutamine (Gibco), nonessential amino acids (Gibco), and sodium pyruvate (Gibco) at 37°C and 5% CO_2_. All cell lines tested negative for mycoplasma contamination (Lonza).

### Virus stocks.

Recombinant LCMV containing an mCherry reporter (rLCMV-mCherry) was rescued from BHK-21 cells transfected with plasmids encoding viral proteins and reporter containing recombinant genome as previously described (30). Both rLCMV-mCherry and LCMV strain ARM-4 (gift of Michael J. Buchmeier) were propagated on BHK-21 cells. Clarified supernatants were collected 48 hpi and stored at −80°C. Viral titers were determined by focus assay on Vero cells. Briefly, serial 10-fold dilutions of virus stocks were used to infect cells in 96-well plates and incubated for 24 h. Infected cells were fixed with 4% paraformaldehyde (PFA), permeabilized with 0.2% Tween 20, and stained with anti-LCMV-NP antibody (1.1.3) (58) and anti-mouse secondary (Alexa Fluor 488; Thermo Fisher Scientific), followed by focus counting. Antibody details can be found in Table S3 in the supplemental material. All experiments with LCMV or recombinant LCMV were performed in a biosafety level 2 laboratory.

10.1128/mbio.00205-22.9 TABLE S3 Antibodies used in this study. Antibodies and their respective dilutions that were used in this study are listed along with their vendor and catalog numbers. Download Table S3, XLSX file, 0.01 MB. Copyright © 2022 Liu et al. 2022 Liu et al. https://creativecommons.org/licenses/by/4.0/ This content is distributed under the terms of the Creative Commons Attribution 4.0 International license.

### Genome-wide CRISPR screen.

A549 cells were stably lentivirally transduced with Cas9-BLAST (Addgene number 52962; gift from Feng Zhang) and subsequently selected using blasticidin. Next, a total of 300 million A549-Cas9 cells were transduced with the lentiviral human GeCKO v2 library (Addgene number 1000000049; gift from Feng Zhang) (29) at an MOI of 0.5 and selected using puromycin for 6 days. To conduct the host factor screen, 120 million (60 million each of sublibraries A and B) A549-Cas9-BLAST GeCKO library cells were infected with rLCMV at an MOI of 10. At 24 hpi, cells that remained mCherry negative were collected using a Sony SH800 cell sorter. Simultaneously, 120 million cells of uninfected A549-Cas9-BLAST GeCKO library cells were collected to assess sgRNA representation as a reference.

Genomic DNA (gDNA) was extracted using the NucleoSpin blood kit (Macherey-Nagel). The sgRNA expression cassettes were amplified from gDNA in a two-step nested PCR using Q5 high-fidelity 2× master mix (NEB). For PCR-I, 48 reaction mixtures (for control samples) and 12 to 24 reaction mixtures (for mCherry-negative sorted fluorescence-activated cell sorting samples) containing 1 μg were amplified for 16 cycles. Reaction mixtures were pooled, mixed, and size selected using SPRIselect (Beckman Coulter). During PCR-II, 10 reaction mixtures containing 5 μL of PCR-I product were amplified for 10 cycles using indexed Illumina primers. PCR products were cleaned using AmpureXP beads (Beckman Coulter) and sequenced on an Illumina NextSeq 500 using a custom sequencing primer. Primer sequences can be found in Table S4.

10.1128/mbio.00205-22.10 TABLE S4 Oligonucleotides used in this study. This table lists all oligonucleotides used in this study for conducting the whole-genome CRISPR screen, for producing sgRNA and genotyping resulting knockout cell lines, for cloning cDNA of interest, and for cloning mammarenavirus glycoprotein-pseudotyped VSV of interest. Download Table S4, XLSX file, 0.01 MB. Copyright © 2022 Liu et al. 2022 Liu et al. https://creativecommons.org/licenses/by/4.0/ This content is distributed under the terms of the Creative Commons Attribution 4.0 International license.

Demultiplexed FASTQ files were aligned to a reference table containing sgRNA sequences, and the abundance of each sgRNA was determined for the starting and sorted cell population. Guide count tables were further processed using MAGeCK to determine positive enrichment scores for each gene (31). Gene ontology enrichment was determined with statistical overrepresentation test on PANTHER (38) using genes from the 300 highest MAGeCK scores.

### Generation of monoclonal KO cell lines.

sgRNA sequences against gene targets were designed using CRISPick (59), and the corresponding DNA oligonucleotides (IDT) were annealed and ligated into pX458 (Addgene number 48138; gift from Feng Zhang) (60). Cells were transfected with pX458 constructs using *Trans*IT-X2 (Mirus Bio), and GFP-positive cells were sorted into 96-well plates using a FACSAria II (BD) 2 days later. Clonal populations were genotyped by Sanger sequencing of the PCR-amplified sgRNA-targeted sites in the gDNA extracted using DNA QuickExtract (Lucigen). Resulting sequences were compared to references, and clones containing a frameshift indel were selected. To determine cell growth of A549 WT and KO cell lines, CellTiter-Glo (Promega) was mixed 1:1 with cells seeded in 96-well plates for three consecutive days, and the luminescence signal was quantified using the GloMax-Multi microplate reader (Promega). A list of all used sgRNA sequences and genotyping primers can be found in Table S4.

### Plasmids, cloning, and lentivirus production.

Human CD164 (number RC202234; Origene) and mouse Cd164 (number MR201951; Origene) cDNAs were cloned into EcoRV-cut plenti-CMV-Puro-DEST (Addgene number 17452; gift from Eric Campeau and Paul Kaufman) (61) using NEBuilder HiFi DNA assembly master mix (NEB). Primers used to assemble expression plasmids for domain deletion mapping and alanine scanning mutagenesis of CD164 can be found in Table S4.

Lentivirus was produced in HEK293T cells by cotransfection of cDNA containing lentiviral plasmid together with helper plasmids pMD2.G (Addgene number 12259; gift from Didier Trono) and pCMV-dR8.91 (Life Science Market) using *Trans*IT-Lenti (Mirus Bio). Supernatants were collected 48 h posttransfection, filtered, and added to recipient cells in the presence of Polybrene (EMD Millipore). Transduced cells were subsequently selected using puromycin (Thermo Fisher Scientific) during days 3 to 5.

### Compound inhibition and antibody neutralization.

Bafilomycin A_1_, bafilomycin B_1_ (Cayman Chemical Company), and concanamycin A (Santa Cruz Biotechnology) were resuspended in dimethyl sulfoxide (DMSO) and stored at −20°C until use. Cells were incubated with compounds for 1 h at 37°C prior to infection assay.

Antibody neutralization assays were conducted by preincubating cells with anti-CD164 clone N6B6 (BD Pharmingen) or mouse IgG isotype control (BD Pharmingen) for 1 h at 37°C prior to infection assay. Antibody details can be found in Table S3.

### Generation of arenavirus pseudotyped vesicular stomatitis virus.

Glycoprotein from LASV (GenBank accession no. AAA46286.1), GTOV (GenBank accession no. AAN05423.1), MACV (GenBank accession no. AIG51558.1), and LCMV strain WE-HPI (Addgene number 15793; gift from Miguel Sena-Esteves) (62) were cloned into a pCAGGS vector backbone using NEBuilder HiFi DNA assembly master mix (NEB). To generate an LCMV strain Cl13-GP (GenBank accession no. DQ361065.2) expression plasmid, mutations N176D and F260L were introduced into pCAGGS-LCMV-Arm4-GP using site-directed mutagenesis. To generate LCMV strain WE54-GP (GenBank accession no. AJ297484.1), mutations V94A, S133T, Y155H, and T211A were introduced into LCMV strain WE-HPI-GP. To generate LCMV strain WE2.2-GP (GenBank accession no. AJ318512.1), mutation S153F was introduced into LCMV strain WE54-GP (22, 23). A list of primers used for cloning and site-directed mutagenesis can be found in Table S4.

To rescue the various VSV-ΔG-Arenavirus-GP pseudotyped virus, 293T cells were transfected with arenavirus glycoprotein expression plasmids using *Trans*IT-LT1 (Mirus Bio). Cells were transduced the following day with VSV-ΔG-GFP (Kerafast) (45) at an MOI of 3 and incubated in medium containing anti-VSV-G antibodies (Kerafest) for 24 h. Clarified supernatants containing pseudovirus were collected and stored at −80°C. Stock titers were measured using flow cytometry on a FACSCelesta (BD). All experiments with pseudotyped VSV were performed in a BSL-2 laboratory.

### Flow cytometry analysis of viral infection assays.

Cells plated in 96-well plates were infected with rLCMV-mCherry or LCMV at an MOI of 1 for an adsorption period of 1 h at 37°C and subsequently cultured for 24 h. To analyze percent infected, cells were trypsinized and fixed in suspension with 4% PFA for 30 min. For infection with rLCMV-mCherry, analysis was done by flow cytometry on FACSCelesta (BD), where approximately 5,000 cells were recorded and gated based on SFC/SSC, FSC-H/FSC-A (singlets), and phycoerythrin-CF594 (mCherry) using FlowJo 10. For infection with LCMV, cells were permeabilized and stained for LCMV-N protein (primary, 113; secondary, Alexa Fluor 488) prior to flow gating for fluorescein isothiocyanate (FITC). Antibody details can be found in Table S3.

For pseudotype infection assays, cells seeded in a 96-well plate were infected with various VSV-Arenavirus-GP pseudoviruses. At 24 hpi, cells were lifted using Tryple Select Enzyme (Gibco) and flowed on a FACSCelesta (BD) and used for FITC (enhanced green fluorescent protein) signal as previously described.

### Indirect immunofluorescence of human placenta.

Five-micron cryosections of human placenta were postfixed, permeabilized, blocked, and stained using standard protocols (53). Briefly, primary CD164 mouse anti-human clone N6B6 (BD Pharmingen), mouse isotype (BD Pharmingen), or cytokeratin-7 rat anti-human (Sigma Millipore) were used to probe sections overnight at 4°C. Immunofluorescence was detected using fluoroconjugated antibodies at a 1:1,000 dilution for 1 h at room temperature. Nuclei were stained with NucBlue (Thermo Fisher), and slides were mounted in ProLong Gold antifade reagent (Thermo Fisher) according to the manufacturer’s instructions. Slides were visualized using a Nikon Ti2 inverted fluorescence microscope with a Crest large-field-of-view spinning disk confocal microscope (CrestOptics). Antibody details can be found in Table S3.

### Western blotting.

Cells were scraped and lysed in radioimmunoprecipitation assay (RIPA) buffer on ice. All lysates were separated by SDS-PAGE on precast 4 to 12% Bis-Tris gels (Thermo Fisher) in the NuPAGE electrophoresis system. Proteins were transferred onto nitrocellulose membrane using the Bio-Rad Mini-Protean Mini Trans-Blot transfer system. Membranes were blocked with Tris-buffered saline with 0.05% Tween 20 and 5% nonfat milk and incubated with primary antibody diluted in blocking buffer overnight at 4°C on a shaker. Primary antibodies were detected by incubating membranes with a 1:15,000 dilution of IRDye secondary antibodies (LI-COR) for 1 h at room temperature and visualized using Odyssey CLx (LI-COR). Antibody details can be found in Table S3.

### Quantification and statistical analysis.

For viral infection, drug treatment, antibody neutralization, and cell growth experiments, biological replicates are defined as independent treatments and measurements from cells harvested from multiple wells on different days. Replicates are displayed as means ± standard errors of the means (SEM) and visualized using GraphPad Prism 9. Dose-response curves for drug treatments and antibody neutralizations were generated by applying a nonlinear curve fit with least-squares regression and default parameters using GraphPad Prism 9. For all experiments, the statistical details can be found in the figure legends.

### Data availability.

Raw sequencing reads can be found in NIH BioProject under accession number PRJNA806912.
