# Supplementary figures and images for: Genome-Wide Knockout Screen Identifies Human Sialomucin CD164 as an Essential Entry Factor for Lymphocytic Choriomeningitis Virus
Source: mBio. 2022 May 3;13(3):e00205-22. doi: 10.1128/mbio.00205-22 (PMC9239079; doi:10.1128/mbio.00205-22)

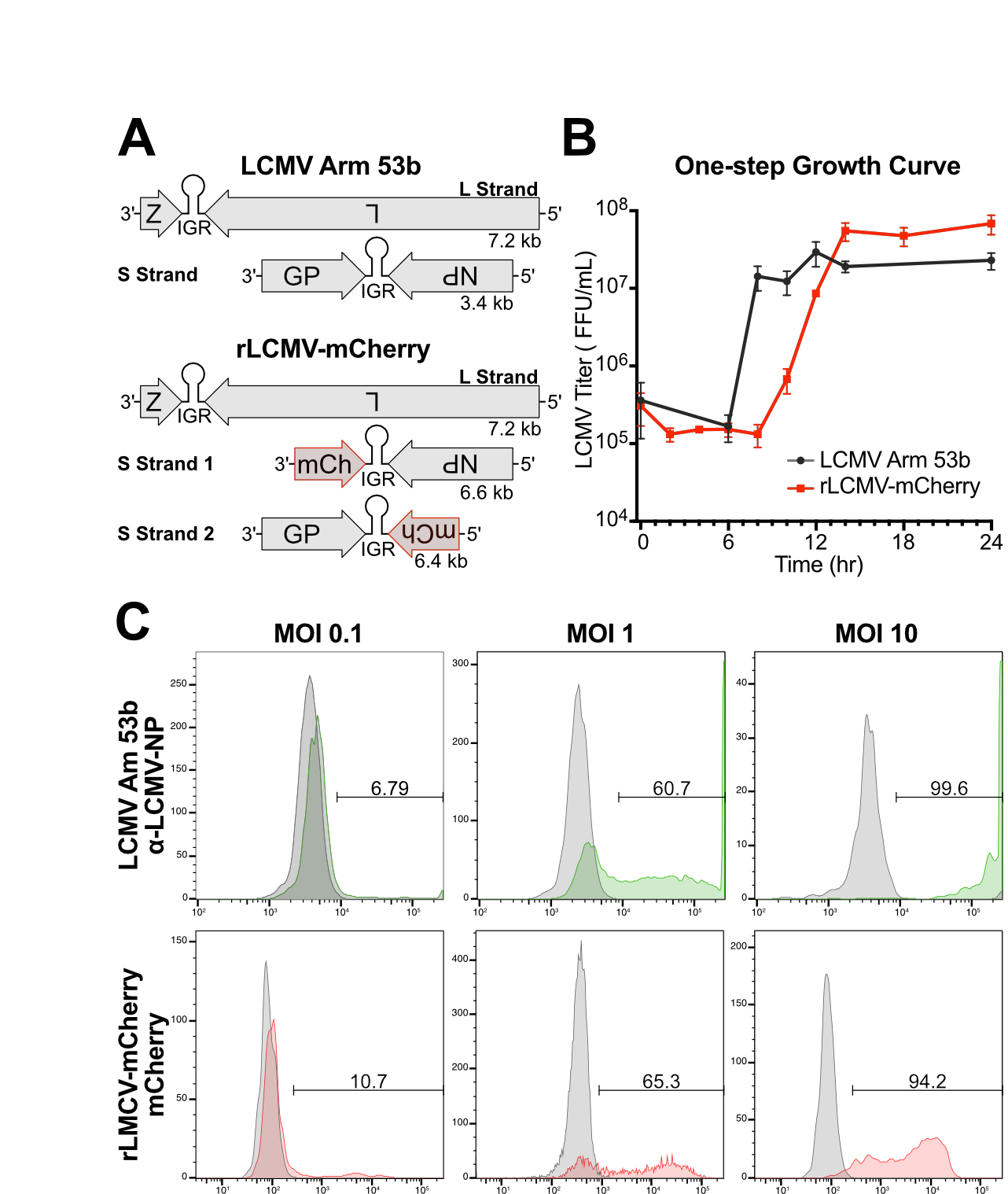

Supplement: FIG S1 [file mbio.00205-22-s0001.tif]

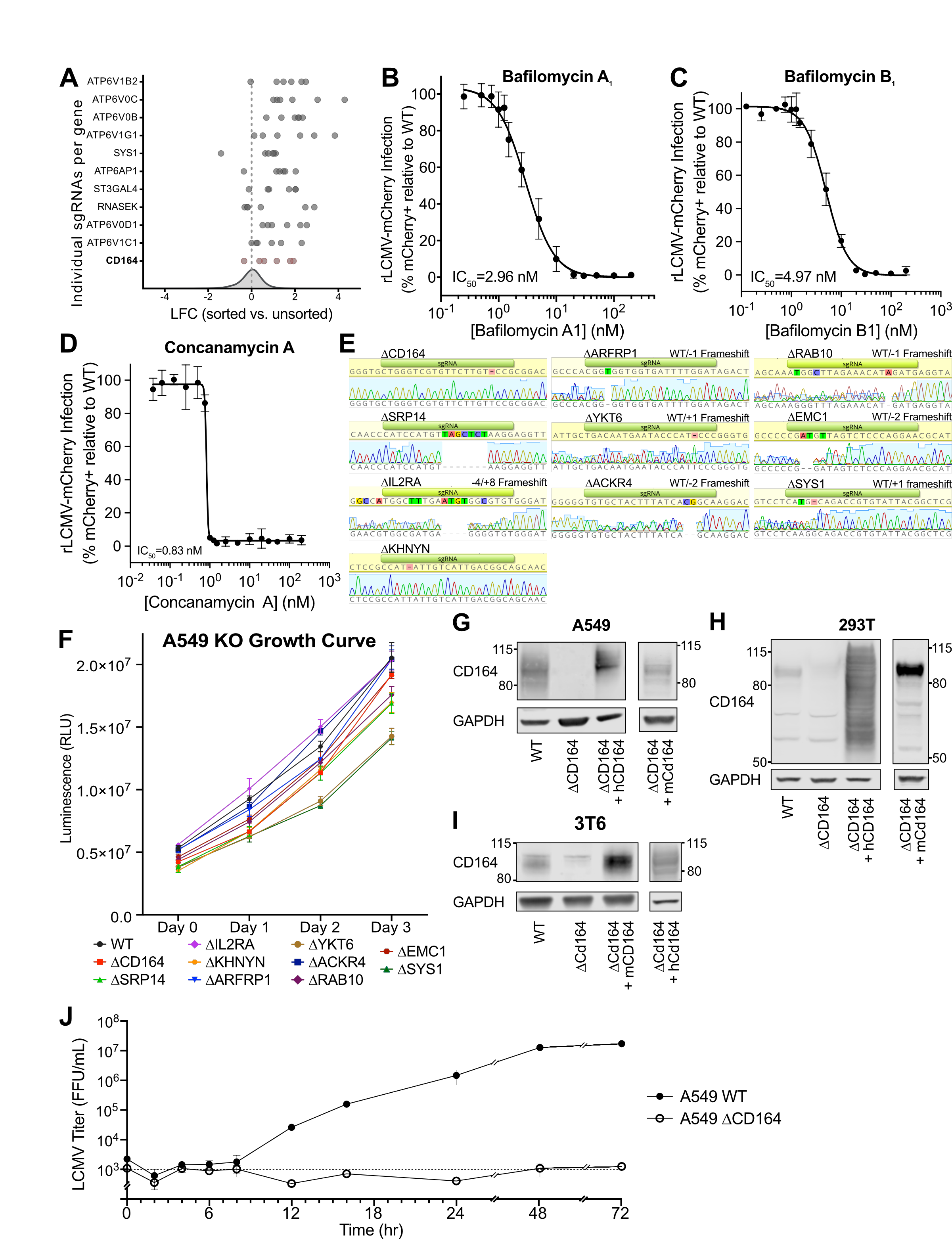

Supplement: FIG S2 [file mbio.00205-22-s0002.tif]

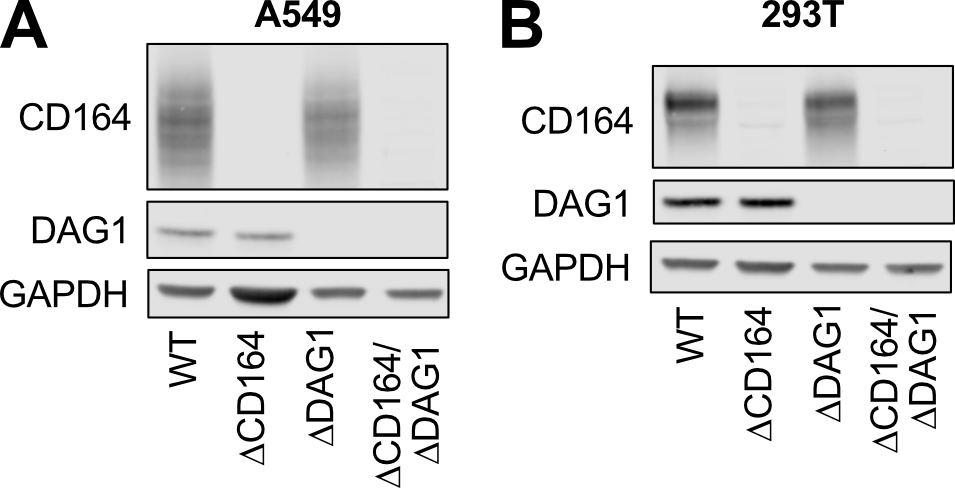

Supplement: FIG S3 [file mbio.00205-22-s0003.tif]

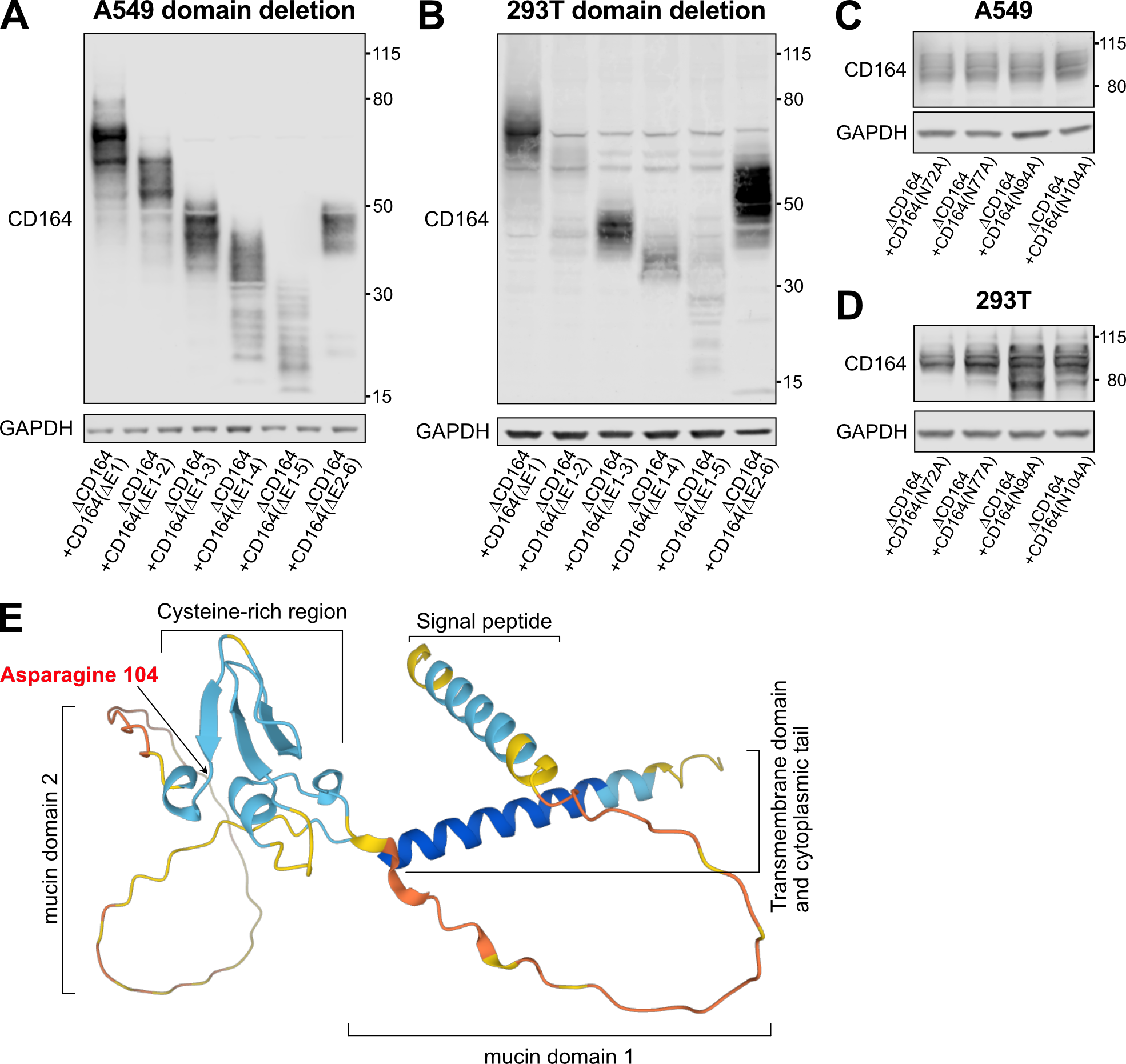

Supplement: FIG S4 [file mbio.00205-22-s0004.tif]

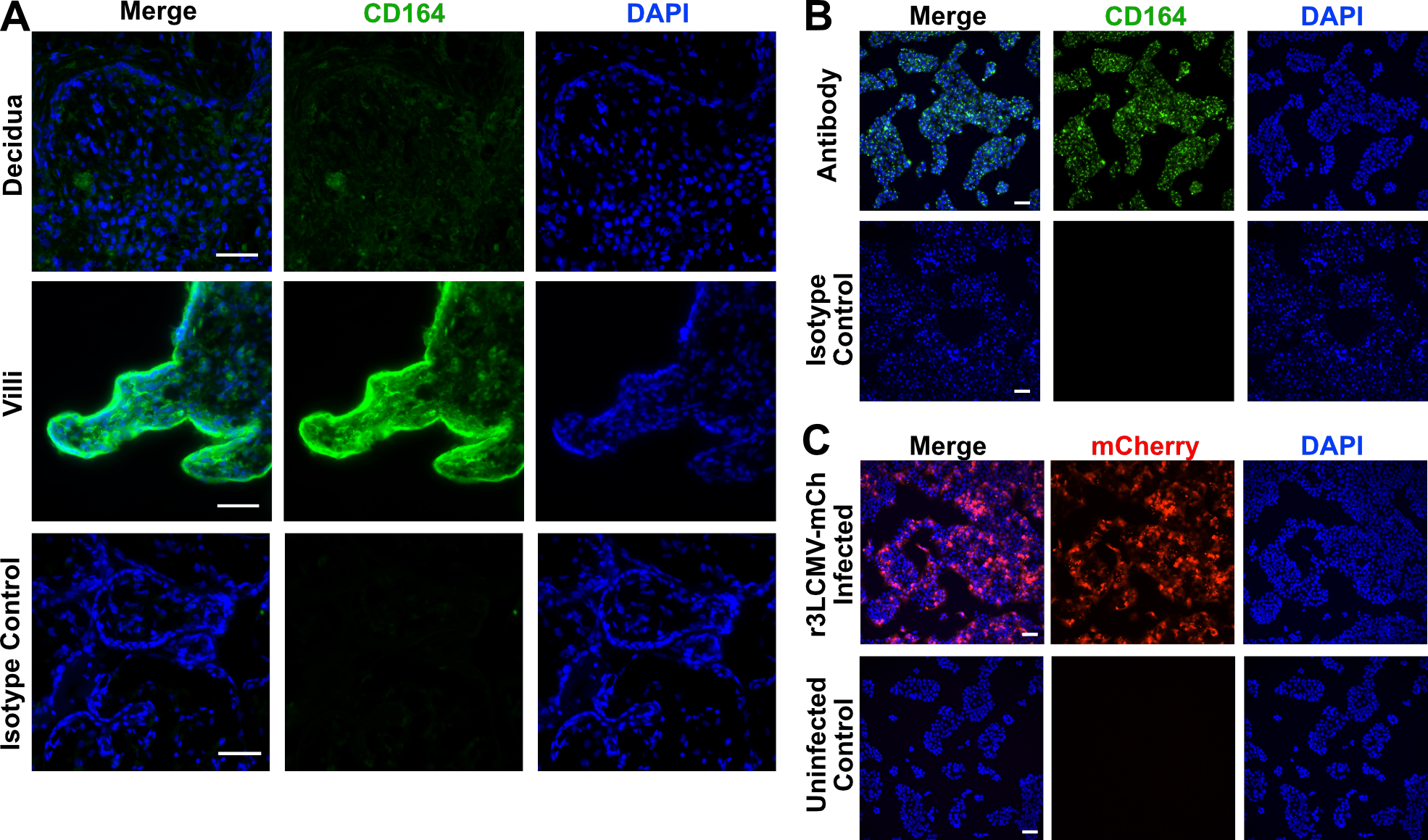

Supplement: FIG S5 [file mbio.00205-22-s0005.tif]

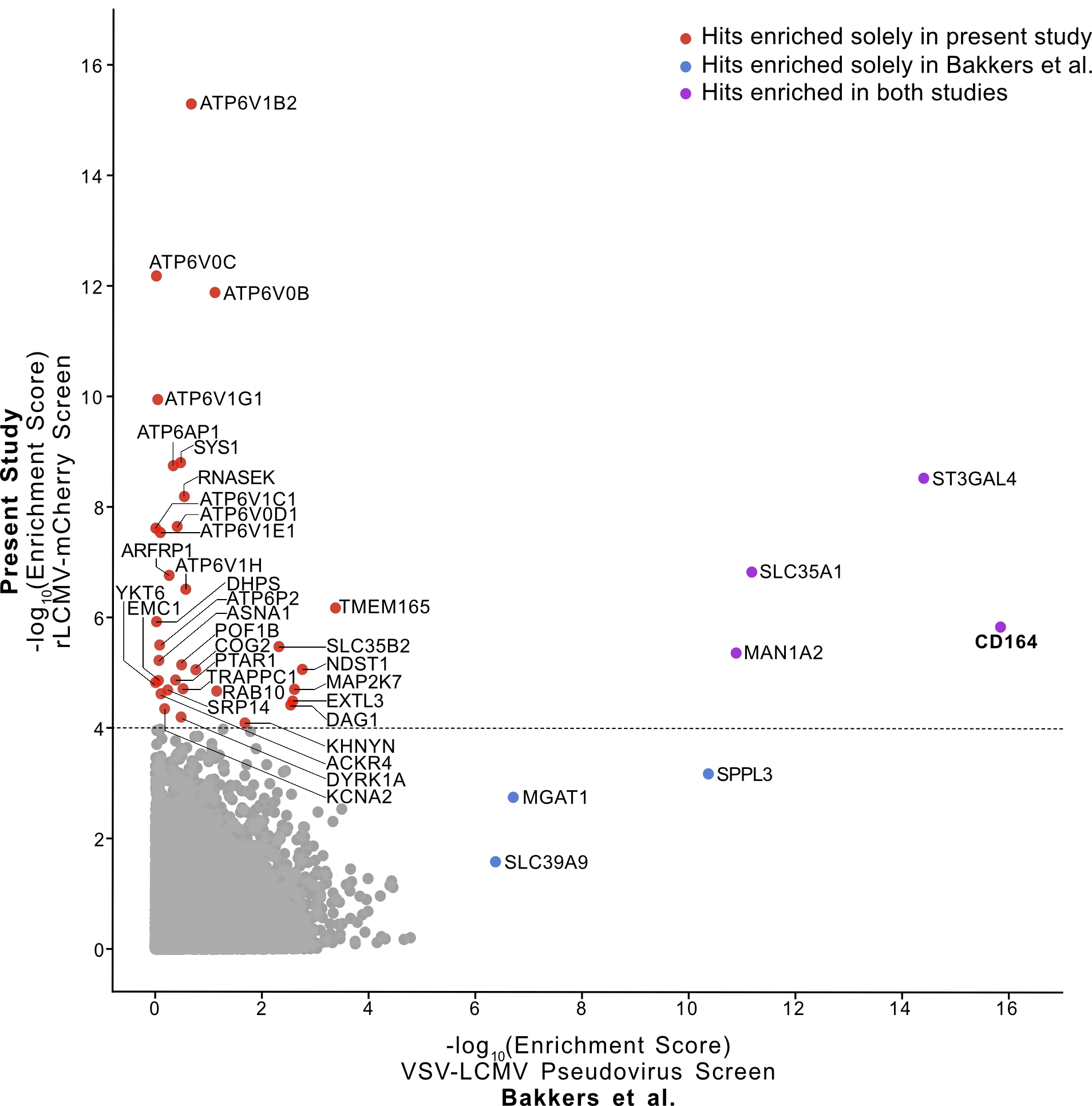

Supplement: FIG S6 [file mbio.00205-22-s0006.tif]
